# Hepatic Stellate Cell Regulation of Liver Regeneration and Repair

**DOI:** 10.1002/hep4.1628

**Published:** 2020-11-13

**Authors:** Laura J. Kitto, Neil C. Henderson

**Affiliations:** ^1^ Centre for Inflammation Research The Queen’s Medical Research Institute Edinburgh BioQuarter University of Edinburgh Edinburgh United Kingdom; ^2^ MRC Human Genetics Unit Institute of Genetics and Molecular Medicine University of Edinburgh Edinburgh United Kingdom

## Abstract

The hepatic mesenchyme has been studied extensively in the context of liver fibrosis; however, much less is known regarding the role of mesenchymal cells during liver regeneration. As our knowledge of the cellular and molecular mechanisms driving hepatic regeneration deepens, the key role of the mesenchymal compartment during the regenerative response has been increasingly appreciated. Single‐cell genomics approaches have recently uncovered both spatial and functional zonation of the hepatic mesenchyme in homeostasis and following liver injury. Here we discuss how the use of preclinical models, from in vivo mouse models to organoid‐based systems, are helping to shape our understanding of the role of the mesenchyme during liver regeneration, and how these approaches should facilitate the precise identification of highly targeted, pro‐regenerative therapies for patients with liver disease.

## Graphical abstract

The hepatic mesenchyme has been studied extensively in the context of liver fibrosis; however, much less is known regarding the role of mesenchymal cells during liver regeneration. Single‐cell genomics approaches have recently uncovered both spatial and functional zonation of the hepatic mesenchyme in homeostasis and following liver injury. Here we discuss how the use of preclinical models are helping to shape our understanding of the role of the mesenchyme during liver regeneration, and how these approaches should facilitate the precise identification of highly targeted, pro‐regenerative therapies for patients with liver disease.

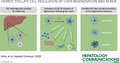

**Abbreviations**
3D: three‐dimensional
aHSC: activated HSC
APAP: acetaminophen
ECM: extracellular matrix
EGF: epidermal growth factor
FGF: fibroblast growth factor
Foxf1: forkhead box protein F1
HGF: hepatocyte growth factor
HSC: hepatic stellate cell
KO: knockout
LSEC: liver sinusoidal endothelial cell
MFB: myofibroblast
PDGF: platelet‐derived growth factor
PHx: partial hepatectomy
scRNA‐seq: single‐cell RNA sequencing
TGF‐β: transforming growth factor beta
VEGF: vascular endothelial growth factor
α‐SMA: alpha smooth muscle actin

The liver has a unique ability to regenerate following injury. Tissue damage provokes a rapid regenerative response aimed at restoration of liver mass and function.^(^ ^1^, ^2^, ^3^ ^)^ However, in many cases of acute and chronic liver disease, this regenerative capacity is overwhelmed. In this setting, liver transplantation is the only curative therapy; however, this approach is limited by shortage of donor organs, high costs, and the requirement for lifelong immunosuppression following transplantation. Although there have been vast improvements in mortality in other chronic diseases over the past 50 years, mortality rates from liver disease have increased exponentially and it is now the third most common cause of premature death in the United Kingdom.^(^ ^4^ ^)^ A greater understanding of the cellular and molecular mechanisms underpinning liver regeneration is required to allow the development of novel and effective pro‐regenerative therapies.

## Cellular Composition of the Hepatic Mesenchyme

**FIG. 1:**
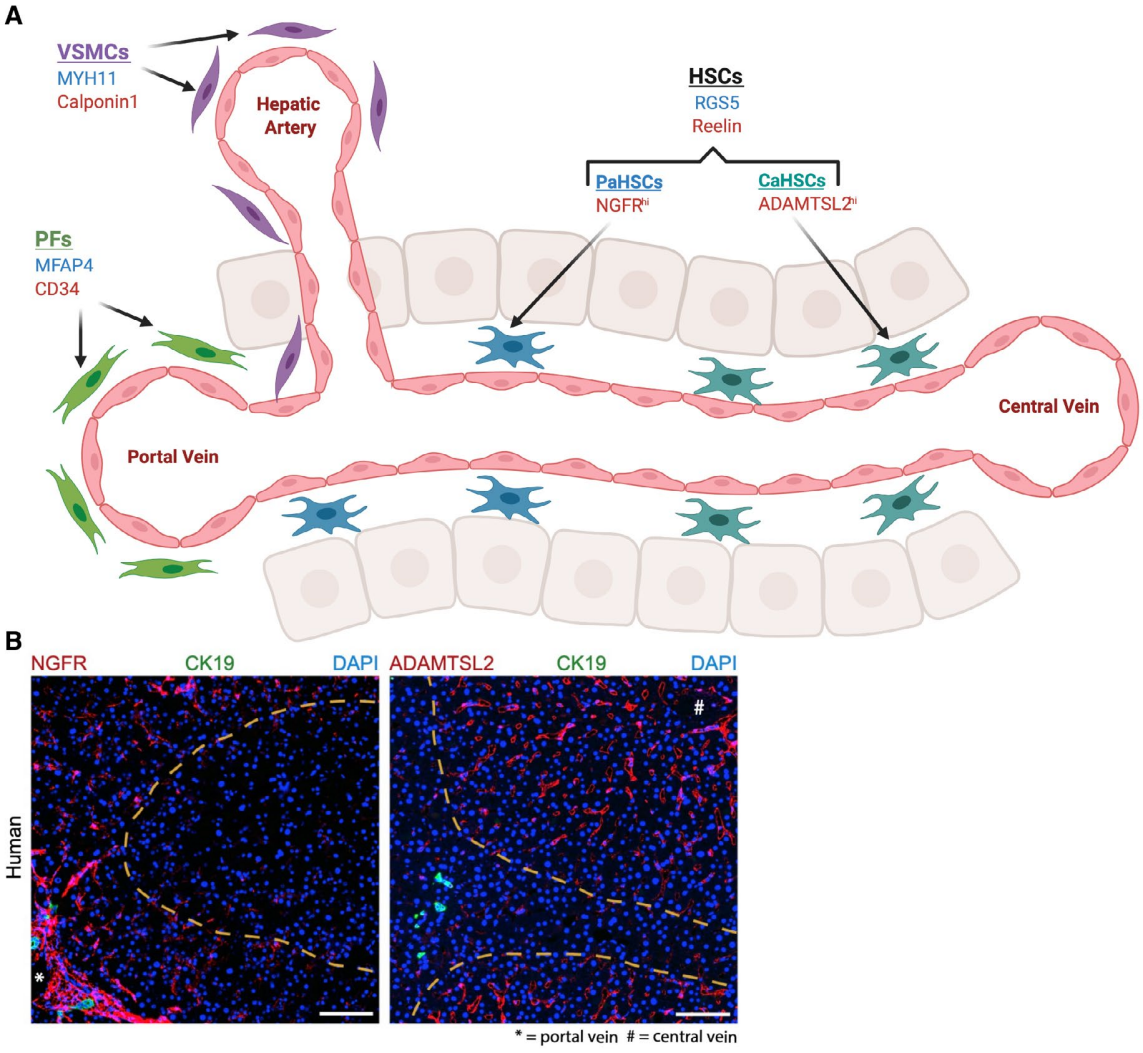
Mesenchymal cell heterogeneity and zonation across the hepatic lobule. A) scRNA‐seq experiments have identified three distinct mesenchymal cell populations in homeostatic liver (HSC, hepatic stellate cells; VSMC, vascular smooth muscle cells; PF, portal fibroblasts), with specific markers in human liver (blue) and mouse liver (red). Furthermore, spatial and functional zonation of HSCs across the hepatic lobule has been identified, with HSCs partitioning into portal vein associated HSCs (PaHSCs) and central vein associated HSCs (CaHSCs), again with specific markers. B) Immunofluorescence image of healthy human liver (Dobie et al.^(^ ^5^ ^)^) demonstrates NGFR positive (red) HSCs around the portal tract (biliary epithelium, green, identified with CK19 staining) and ADAMTSL2 positive (red) HSCs around the central vein. Scale bar, 100 μm. Abbreviations: MYH11, Myosin heavy chain 11; NGFR, Nerve growth factor receptor.

Single‐cell genomics approaches are transforming our understanding of disease pathogenesis across hepatology, allowing interrogation of cell populations in health and disease at unprecedented resolution.^(^ ^5^, ^6^, ^7^, ^8^, ^9^, ^10^ ^)^ Recently, single‐cell RNA sequencing (scRNA‐seq) experiments have enabled deconvolution of the mouse hepatic mesenchyme, confirming and further characterizing three distinct mesenchymal subpopulations: portal fibroblasts (residing in the portal niche), vascular smooth muscle cells (residing within the hepatic artery and portal vein walls), and hepatic stellate cells (HSCs, located in the perisinusoidal space throughout the parenchyma) (Fig. 1).^(^ ^5^ ^)^

HSCs are a major mesenchymal cell type, consisting of approximately one third of nonparenchymal cells and 8% of all cells in the homeostatic liver.^(^ ^11^ ^)^ They are located in the perisinusoidal space of Disse between the fenestrated sinusoidal endothelial cell layer and the hepatic epithelial cells (hepatocytes). The space of Disse contains connective tissue matrix, which provides cellular support and signals to maintain the differentiated function of HSCs and allows unimpeded transport of solutes and growth factors.^(^ ^12^, ^13^ ^)^

## HSC Function in Homeostasis

HSCs in the homeostatic liver are “quiescent” and are characterized by long dendritic cytoplasmic processes and storage of vitamin A (retinol).^(^ ^14^ ^)^ These cytoplasmic processes facilitate direct contact with liver sinusoidal endothelial cells (LSECs), hepatocytes, and Kupffer cells, allowing intercellular cross‐talk and transport of soluble mediators and cytokines. Microprojections on the surface of the cytoplasmic processes detect chemotactic signals, and along with the expression of a large number of receptors and mediators that modulate cellular contraction,^(^ ^15^, ^16^ ^)^ allow HSCs to regulate sinusoidal tone and blood flow.^(^ ^17^ ^)^ Under physiological conditions, HSCs regulate extracellular matrix (ECM) turnover in the space of Disse through the secretion of ECM proteins, degrading enzymes (matrix metalloproteinases), and their tissue inhibitors (tissue inhibitors of metalloproteinases).^(^ ^18^ ^)^ Quiescent HSCs also produce a range of growth factors and other mediators, including hepatocyte growth factor (HGF, the most potent mitogen for hepatocytes) and vascular endothelial growth factor (VEGF, a mitogen for sinusoidal and endothelial cells).^(^ ^16^ ^)^

HSCs express a variety of markers that have been used to distinguish them from other liver cell types. Traditional widely accepted markers included lecithin‐retinol acyltransferase (Lrat), desmin, glial fibrilliary acidic protein (Gfap, quiescent state), and alpha smooth muscle actin (α‐SMA, activated state).^(^ ^11^, ^13^, ^19^ ^)^ Studying the mesenchyme at unprecedented resolution with scRNA‐seq has allowed deeper interrogation of these traditional HSC markers—some of which have now been shown to be broader in their coverage of mesenchymal populations than previously thought (e.g., desmin)—and has enabled the discovery of highly specific HSC markers such as Reelin in mice^(^ ^5^ ^)^ and RGS5 in humans.^(^ ^10^ ^)^

## HSC Activation

In the classical paradigm, following injury, HSCs become activated to ECM‐secreting myofibroblast (MFB)‐like cells.^(^ ^18^, ^19^, ^20^, ^21^ ^)^ *In vitro* studies have suggested that HSC activation is accompanied by loss of retinoid droplets, although this may not be the case in the *in vivo* setting, and the significance of retinoid loss in HSC remains unclear.^(^ ^22^, ^23^ ^)^ Activated HSCs (aHSCs) lay down ECM to produce a temporary scar at the site of injury and help protect against ongoing damage.

HSCs were traditionally considered to be a functionally homogeneous population, all with equal propensity to transition to the activated, collagen‐secreting MFB phenotype following injury. However, a recent scRNA‐seq study has demonstrated spatial and functional zonation of HSCs across the hepatic lobule, identifying portal vein–associated HSCs and central vein–associated HSCs, with the latter responsible for most of the collagen production following induction of centrilobular liver injury (Fig. 1).^(^ ^5^ ^)^

**FIG. 2:**
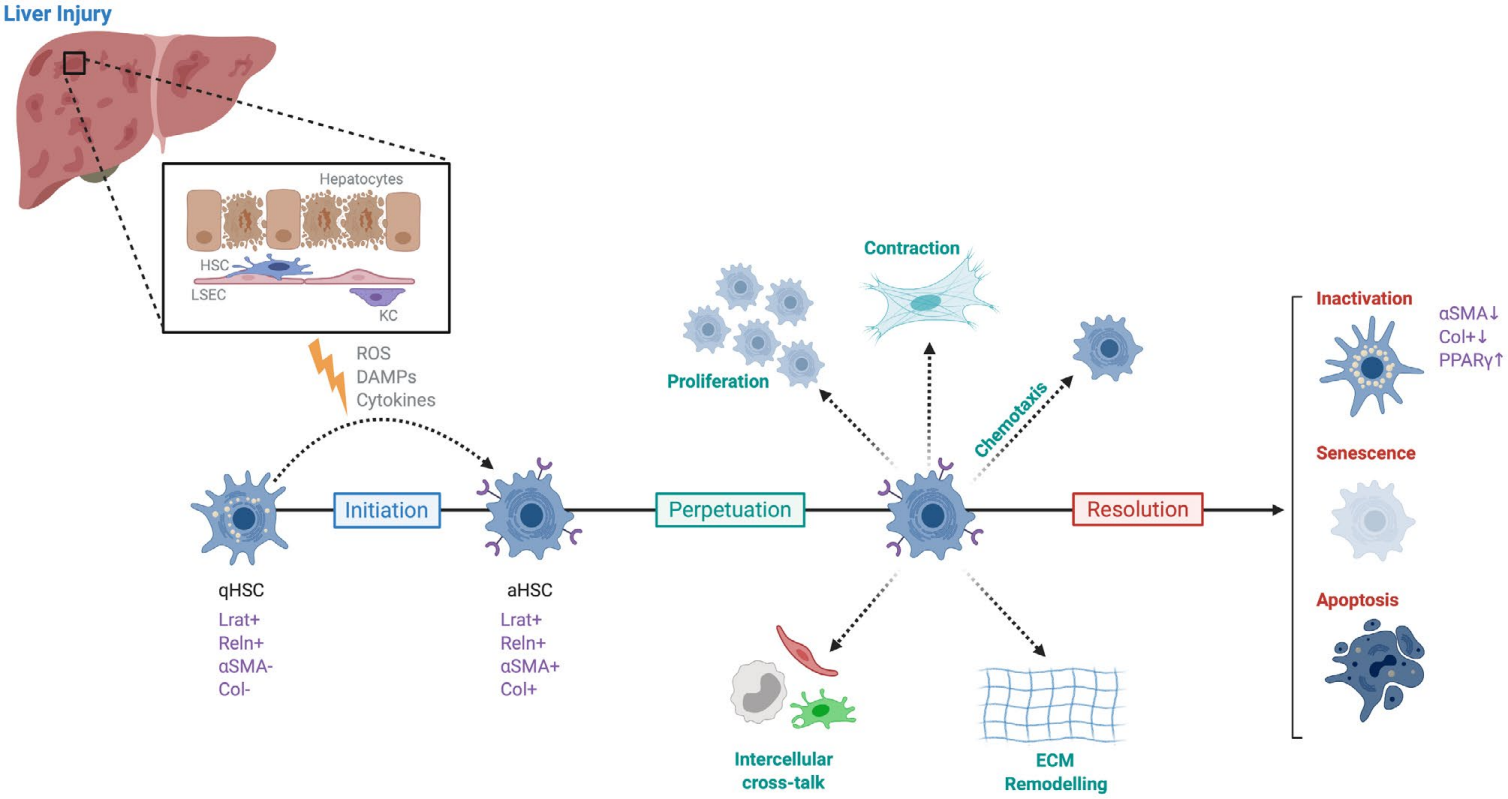
Phases of hepatic stellate cell activation and resolution. Initiation of hepatic stellate cell (HSC) activation occurs following liver injury, and is driven by a variety of signals^(^ ^19^ ^)^ including reactive oxygen species (ROS), damage associated molecular patterns (DAMPs) and cytokines released from damaged hepatocytes. During the initiation phase, quiescent hepatic stellate cells (qHSCs) transdifferentiate to their activated phenotype (aHSC). The perpetuation phase follows, characterised by a range of HSC phenotypic changes. When injury has subsided, a resolution phase follows, where HSCs undergo apoptosis, become senescent or revert to an inactive phenotype, which is more responsive to subsequent injurious stimuli. Abbreviations: LSEC, liver sinusoidal endothelial cell; KC, kupffer cell; Reln, reelin.

HSC activation is a complex, tightly regulated response to injury and proceeds along a continuum, involving progressive changes in cellular function.^(^ ^12^ ^)^ “Initiation” of HSC activation is driven by the injury‐induced influx of inflammatory cells and alterations in ECM composition. HSCs undergo changes in gene expression and phenotype, rendering them increasingly responsive to cytokines and other local stimuli.^(^ ^19^, ^24^ ^)^ Initiation is followed by a “perpetuation” phase; during which the activated HSC phenotype is amplified. This phase involves proliferation, contraction, chemotaxis, altered matrix degradation, and cross‐talk with inflammatory cells.^(^ ^19^, ^24^ ^)^ Finally, provided the injurious stimuli is no longer present, a “resolution” phase follows (Fig. 2). Although HSCs have been studied extensively in the context of homeostasis and hepatic fibrosis, much less is known about their role in the hepatic regenerative response.

## Preclinical Models of Acute Liver Injury and Regeneration

Regeneration is the ability to recreate original tissue architecture and function following injury, without leaving a scar.^(^ ^1^ ^)^ In the clinical setting, liver regeneration can be observed in any condition, resulting in loss of hepatocytes, including viral, toxic (alcohol, metabolic diseases), ischemic, or autoimmune conditions. Although there is very little cell division during homeostasis, the liver has an extraordinary ability to regenerate following an acute insult, with hepatocyte proliferation re‐establishing homeostasis within days.^(^ ^1^ ^)^ This is highly dependent on cross‐talk between hepatocytes and nonparenchymal cells. Preclinical models have long been used to understand the mechanisms underlying liver regeneration, with transgenic rodent models providing important insights into the role of the mesenchyme in the regenerative response. The models used to study liver regeneration can be broadly grouped into three main categories: surgical resection (partial hepatectomy), chemical injury models, and organoid models.

### PARTIAL HEPATECTOMY MODEL OF LIVER REGENERATION

Partial hepatectomy (PHx) is one of the oldest and most commonly used preclinical models for the study of liver regeneration and was first described by Higgins and Anderson in the 1930s. Two‐thirds of the rodent liver is surgically removed, prompting a hyperplastic response in the remaining structurally intact lobes, restoring original liver mass, usually within 7 days following surgery.^(^ ^25^, ^26^, ^27^ ^)^ This primarily occurs through the proliferation and transdifferentiation of mature cells, which switch from a quiescent to proliferative state and re‐enter the cell cycle.^(^ ^28^, ^29^ ^)^ PHx is a simple, reproducible, and highly tractable model with which to study the cellular mechanisms regulating liver regeneration and has allowed detailed interrogation of the regenerative response. The multilobular structure of the mouse liver enables removal of the anterior lobes without significant necrosis or inflammation in the residual tissue,^(^ ^30^ ^)^ and the procedure itself is of very short duration, allowing precise analysis of the signaling events during liver regeneration.

In the clinical setting, the PHx model is most relevant to removal of solitary metastatic lesions or resections following trauma. However, although PHx has contributed significantly to our understanding of the mechanisms involved in initiation and termination of liver regeneration, it does not account for tissue necrosis, the immune response, and the varying degrees of acute or chronic inflammation observed during the human regenerative response.^(^ ^31^ ^)^

### CHEMICAL INJURY–INDUCED LIVER REGENERATION

Liver regeneration following toxic injury has been well described for several toxins, including thioacetamide, CCl_4_, allyl alcohol, and acetaminophen (APAP). APAP is the most commonly used over‐the‐counter antipyretic and analgesic drug worldwide,^(^ ^32^ ^)^ and APAP overdose is the most common cause of acute liver failure in the Western world.^(^ ^33^ ^)^ Therefore, studying liver regeneration after APAP overdose has clinical and translational relevance. The mechanisms of APAP‐induced hepatotoxicity have been studied extensively and are well understood. Following ingestion, APAP is metabolized to its reactive metabolite N‐acetyl‐p‐benzoquinone imine. N‐acetyl‐p‐benzoquinone imine is eliminated when conjugated to glutathione, and when cellular stores of glutathione are depleted, it covalently binds to cellular proteins, causing oxidative stress and centrilobular hepatocellular necrosis. Necrotic cells release damage‐associate molecular patterns, resulting in inflammatory cell recruitment,^(^ ^34^ ^)^ and dying hepatocytes release proteolytic enzymes, which exacerbate injury.^(^ ^35^, ^36^ ^)^ Hepatocytes in closest proximity to the necrotic zones divide and replace dead cells, allowing recovery to occur.^(^ ^37^ ^)^ In the case of overwhelming injury, acute liver failure ensues, potentially resulting in multi‐organ failure and death.

APAP‐induced liver injury can be modeled in rodents using a single intraperitoneal dose of APAP. This model is well‐characterized and provides a contrasting regenerative model to PHx, more closely resembling human pathophysiology in terms of injury severity, tissue necrosis, immune response, and recovery.^(^ ^34^, ^38^ ^)^ Following APAP administration, rodents develop extensive centrilobular necrosis, which is followed by a robust regenerative response.^(^ ^37^ ^)^ Liver injury can be assessed by the degree of hepatic necrosis identified histologically and by alanine aminotransferase (ALT) measurement. Complete histological recovery and ALT normalization is usually achieved by 72 hours.

Although preclinical models are highly accessible and tractable methods with which to study the hepatic regenerative response, they are unlikely to mimic all relevant aspects of human liver regeneration. Furthermore, studies have shown that cellular and molecular pathways may vary, depending on the nature of the underlying injury. Although inhibition of the epidermal growth factor receptor almost completely abolished hepatocyte proliferation and impaired survival following APAP,^(^ ^39^ ^)^ regeneration was only delayed (and not prevented) following PHx.^(^ ^40^ ^)^ Such studies highlight that different animal models can provide complementary insights into the regenerative response. The PHx and APAP models of liver regeneration are compared and contrasted in Table 1.

**TABLE 1:** Comparison of Surgical Resection and Chemical Injury Models of Liver Regeneration

	PHx	APAP
Timing of injury	Known time of surgery	Undefined
Hepatocyte proliferation	All hepatocytes	Centrilobular, surrounding necrotic zones
Peak hepatocyte proliferation	48 hours	48 hours
Cell cycle	Synchronous	Unsynchronized
Necrosis	Minimal	Significant, widespread
Inflammatory response	Not significant	Extensive

Note:

Adapted from Bhushan et al. (2019).^(^ ^33^ ^)^

### ORGANOID MODELING OF LIVER REGENERATION

An organoid is defined as an *in vitro* three‐dimensional (3D) cellular cluster derived from primary tissue, embryonic stem cells, or induced pluripotent stem cells, capable of self‐renewal and self‐organization, which recapitulates the functionality of the tissue of origin.^(^ ^41^, ^42^ ^)^ Organoids offer an alternative *in vitro* system with which to study liver regeneration, and a promising model to bridge the translational gaps among 2D cultures, *in vivo* mouse models, and study of the human liver regenerative response. Organoids provide new, experimentally tractable, physiologically relevant models of organ development and human pathologies,^(^ ^41^ ^)^ which in many cases are more malleable in terms of manipulation of the regenerative niche, signaling pathways, and genome editing than *in vivo* models.^(^ ^43^ ^)^

Much of the literature to date has focused on generation of liver organoids, their role in the study of fibrosis and cancer, and far less on liver regeneration. Aloia et al.^(^ ^44^ ^)^ compared the transcriptional profile of cholangiocyte organoids and cholangiocytes isolated from livers of mice given a 3,5‐ diethoxycarbonyl‐1,4‐dihydrocollidine supplemented diet. Similar genome‐wide changes were identified in ductal cells *in vivo* and *in vitro*, suggesting that ductal cells undergo a significant change in their transcriptional landscape in response to tissue damage and validating organoids as a potential model system to facilitate the study of specific mechanistic aspects of tissue regeneration.^(^ ^44^ ^)^ Given the challenges of culturing HSCs *in vitro*, development of mature HSCs from induced pluripotent stem cells would be ideal. However, few groups so far have achieved this^(^ ^45^, ^46^ ^)^: following an already established protocol for the induction of induced pluripotent stem cells to mesoderm, using the surface marker activated leukocyte cell adhesion molecule to select HSC progenitors, and further differentiating these to mature HSCs by inhibiting the Rho signaling pathway.^(^ ^45^ ^)^ Under these conditions, cells acquired HSC morphology, vitamin A storage capabilities, and expressed HSC markers including nerve growth factor receptor, LRAT, and HGF. Further studies are required to confirm the efficacy of this system for disease modelling.

Intestinal organoid cultures have already provided mechanistic insights into epithelial repair following injury. These 3D organoid cultures have significantly deepened our understanding of the regenerative pathways induced following radiation or chemical damage and the biological mechanisms that mediate regeneration of the epithelium.^(^ ^47^ ^)^ Although organoid cultures also offer much promise in the field of liver regeneration, more work is required to establish and refine effective multilineage 3D coculture systems, to allow in‐depth study of the cross‐talk between the epithelial component of liver‐derived organoids and other cell lineages.

## Mesenchymal Cell Dynamics During Liver Regeneration

Minimal cell division occurs during homeostasis in the adult liver, but injury provokes a rapid regenerative response aimed at restoration of liver mass and function.^(^ ^1^, ^2^, ^3^ ^)^ Increased numbers of HSCs are identified in the liver following injury, reflecting both local proliferation and accumulation in regions of injury by chemotaxis.^(^ ^12^ ^)^ Following injury, hepatocytes are the first hepatic cell type to enter DNA synthesis and synthesize several growth factors responsible for inducing proliferation in nonparenchymal cells. These include platelet‐derived growth factor (PDGF; the most potent proliferative and chemoattractant stimulus for HSCs^(^ ^12^ ^)^) and fibroblast growth factors 1 and 2 (FGF1 and 2). Despite their key role in liver regeneration, there is much less data in the current literature regarding the temporal dynamics of the mesenchymal cell proliferative response following acute liver injury; however, the general consensus is that nonparenchymal cells enter DNA synthesis 24 hours after hepatocytes, with peak proliferative activity at 48 hours or later.^(^ ^27^, ^48^, ^49^, ^50^ ^)^

## Mesenchymal Cell Function During Liver Regeneration

As knowledge of the cellular mechanisms driving liver regeneration has increased, the regulatory role of the hepatic mesenchyme during this process has become increasingly appreciated.^(^ ^11^, ^51^, ^52^, ^53^, ^54^ ^)^ HSCs have been shown to have a profound impact on the proliferation, differentiation, and morphogenesis of other hepatic cell types during liver development and regeneration,^(^ ^11^, ^55^, ^56^ ^)^ mediated through production of growth factors and cytokines, as well as remodeling of the ECM.^(^ ^11^ ^)^ Inhibition of HSC activation (using gliotoxin^(^ ^52^ ^)^ and l‐cysteine^(^ ^51^ ^)^) has a significant impact on the regenerative capacity of the liver. Following APAP administration, gliotoxin‐treated mice display increased liver damage, a 66% reduction in hepatocyte proliferation (accompanied by reduced expression of genes usually up‐regulated during their replication, such as HGF and IL‐6), and reduced survival. Similarly, rats maintained on a diet supplemented with l‐cysteine demonstrated significantly less oval cell proliferation following 2‐acetylaminofluorene/PHx. In both cases, aHSCs were deemed to be central to the regenerative process, acting as a major cytokine source to drive regeneration and providing a fibronectin‐rich provisional matrix as a basis for epithelial regeneration. Using the CCl_4_ model of liver injury in forkhead box protein F1 (Foxf1)‐deficient mice, Costa el al. demonstrated that regenerating Foxf1^+/‐^ livers exhibited defective HSC activation.^(^ ^57^ ^)^ Baseline liver injury was comparable in wild‐type and Foxf1^+/‐^ mice, but Foxf1^+/‐^ mice developed more severe pericentral necrosis following CCl_4_ and their survival was impaired, providing further support for a key role of HSCs in the regenerative response.^(^ ^57^ ^)^ Methods of HSC manipulation and their phenotypic effects during the hepatic regenerative response have been summarized in Table 2.

**TABLE 2:** Manipulation of HSCs and Their Phenotypic Effects

	Method of HSC Inhibition and Injury Model	Functional Impact of HSC Targeting
Shen et al. (2011)^(^ ^50^ ^)^	Depletion of activated HSCs with gliotoxin APAP‐induced injury in mice	‐ Reduced aHSCs (↓α‐SMA) ‐ Significantly more necrosis ‐ More infiltrating CD45^+^ cells ‐ 66% decrease in proliferating hepatocytes ‐ Reduced expression of genes usually up‐regulated during liver regeneration (i.e., HGF and EGF)
		
Pintille et al. (2010)^(^ ^49^ ^)^	Inhibition of HSC activation with L‐cysteine. 2AAF/PHx injury model in rats	‐ 11.1‐fold reduction of aHSCs (↓desmin) ‐ Reduction in proliferating cells of all lineages ‐ Reduced oval cell response
		
Kalinichenko et al. (2003)^(^ ^55^ ^)^	Deficient HSC activation in Foxf1‐depleted mice CCl_4_injury	‐ Defective HSC activation (↓α‐SMA, ↓col) ‐ More severe pericentral necrosis and apoptosis ‐ Increased mortality (seems to be somethingwrong with the table formating here ‐ there is greater spacing between the bullet points in this row of the column and they didnt line up!)
		
Passino et al. (2007)^(^ ^52^ ^)^	Defective HSC activation in P75^NTR‐/‐^ mice Crossed with plg‐/‐ mice (spontaneously develop liver disease)	‐ Failure of HSC activation (↓α‐SMA, ↓col) ‐ Reduced hepatocyte proliferation (*in vivo* and *in vitro*) ‐ 2/3 reduction of HGF in liver homogenates ‐ More severe liver disease and reduced survival
		

Abbreviation: 2AAF, 2‐acetylaminofluorene.

### ROLE OF HSC GROWTH FACTORS AND CYTOKINES IN LIVER REGENERATION

HSCs produce a range of growth factors and cytokines that have been shown to drive liver regeneration. Autocrine HSC signaling allows HSCs to tightly regulate the regenerative niche. PDGF is a potent inducer of HSC proliferation, and transforming growth factor beta (TGF‐β) is a potent inducer of the expression of collagen I and other ECM constituents by HSCs.^(^ ^18^, ^58^ ^)^ Both factors, and their corresponding receptors, are expressed during HSC activation.^(^ ^59^, ^60^ ^)^

HSC paracrine signaling also plays an important role during liver regeneration. Chang et al. obtained culture media from culture‐activated HSCs isolated from healthy mouse liver.^(^ ^56^ ^)^ Mice that received systemic infusion of HSC culture media containing HSC‐derived paracrine factors demonstrated a significant survival benefit, with reduced hepatocellular death, increased hepatocyte proliferation, and up‐regulation of liver regeneration–relevant genes following APAP‐induced liver injury. All protective benefits of HSC culture media were abolished by heat inactivation before infusion, providing evidence that HSC‐derived paracrine factors offer trophic support to the liver by inhibiting liver cell death and stimulating regeneration.

One key factor produced by HSCs is HGF. HSCs synthesize HGF on a continual basis as a biologically inactive, single‐chain polypeptide that is stored in the ECM in large quantities. Its receptor (c‐Met) is expressed in hepatocytes, biliary cells, and endothelial cells. HGF has a key role in the initiation of liver regeneration, activating its receptor early following injury and acting as a direct mitogen for hepatocytes. Within the first hour following PHx, there is a significant increase in HGF^(^ ^61^ ^)^ and early activation of the cMet receptor.^(^ ^18^ ^)^ The importance of HSC activation and subsequent HGF production was outlined in a study by Passino et al.,^(^ ^54^ ^)^ which identified the neurotrophin receptor P75^NTR^ as a mediator of this process. HSCs from P75^NTR^‐deficient mice (P75^NTR‐/‐^) failed to adopt an activated phenotype and did not support hepatocyte proliferation. When crossed with plasminogen‐deficient mice, which spontaneously develop liver disease induced by fibrin deposition, Plg double mutant mice exhibited significantly exacerbated liver disease, with reduced HGF production and hepatocyte proliferation. Rho is known to promote the activated state of HSCs,^(^ ^62^ ^)^ and a signaling relationship between Rho and P75^NTR^ is well documented in the nervous system.^(^ ^63^ ^)^ Adenoviral delivery of constitutively activated Rho to P75^NTR^‐deficient HSCs *in vitro* restored activation, prompting the conclusion that P75^NTR^ promotes HSC activation through Rho, and once activated, HSCs secrete HGF to drive hepatocyte proliferation during the regenerative response.

TGF‐β is a multifunctional cytokine with a broad range of effects in homeostasis and regeneration and is produced by HSCs and Kupffer cells. There are three different isoforms of TGF‐β that are present in the liver, all of which bind to the same receptor and are present in all hepatic cell types.^(^ ^64^ ^)^ During peak regeneration, TGF‐β has been shown to drive the production of ECM by HSCs and to promote angiogenesis.^(^ ^65^ ^)^ TGF‐β is mito‐inhibitory in hepatocyte cultures, and following PHx, circulating alpha‐2 macroglobulin binds TGF‐β and transports it to hepatocytes where it is inactivated,^(^ ^27^, ^64^ ^)^ allowing hepatocyte proliferation. HSCs also produce norepinephrine,^(^ ^66^ ^)^ which is known to down‐regulate the mito‐inhibitory effects of TGF‐β ^(^ ^67^ ^)^ and enhance the mitogenic effect of HGF and epidermal growth factor (EGF) in serum‐free hepatocyte cultures.^(^ ^66^ ^)^ Circulating norepinephrine levels increase following PHx, and use of prazosin (a specific A_1_AR antagonist) suppressed hepatocyte DNA synthesis for 3 days after PHx. Similar results were seen following surgical sympathectomy of the liver before PHx.^(^ ^68^ ^)^ Norepinephrine also stimulates production of HGF by mesenchymal cells^(^ ^69^ ^)^ and production of EGF from Brunner’s glands of the duodenum.^(^ ^70^ ^)^

### HSC‐MEDIATED REGULATION OF ANGIOGENESIS

Angiogenesis, the formation of new microvasculature from pre‐existing blood vessels and mature endothelial cells,^(^ ^71^ ^)^ is a hypoxia‐stimulated, growth factor–dependent process that is vital during the regenerative response. HSCs are strategically positioned within the space of Disse to enable cross‐talk with hepatocytes and LSECs, therefore ensuring appropriate vascular growth and integrity during regeneration.^(^ ^11^, ^53^ ^)^ Furthermore, HSCs regulate vessel stabilization and sinusoidal remodeling through direct contact and paracrine interactions with LSECs.^(^ ^72^ ^)^ In particular, PDGF, TGF‐β1, FGF, and VEGF have been shown to exert a potent pro‐angiogenic effect.^(^ ^72^ ^)^ The aHSCs also express angiopoietins, which are important growth factors regulating angiogenesis through receptor tyrosine kinases expressed on LSECs.^(^ ^73^ ^)^

### ECM SCAFFOLD FORMATION AND REMODELING OF ECM

**FIG. 3:**
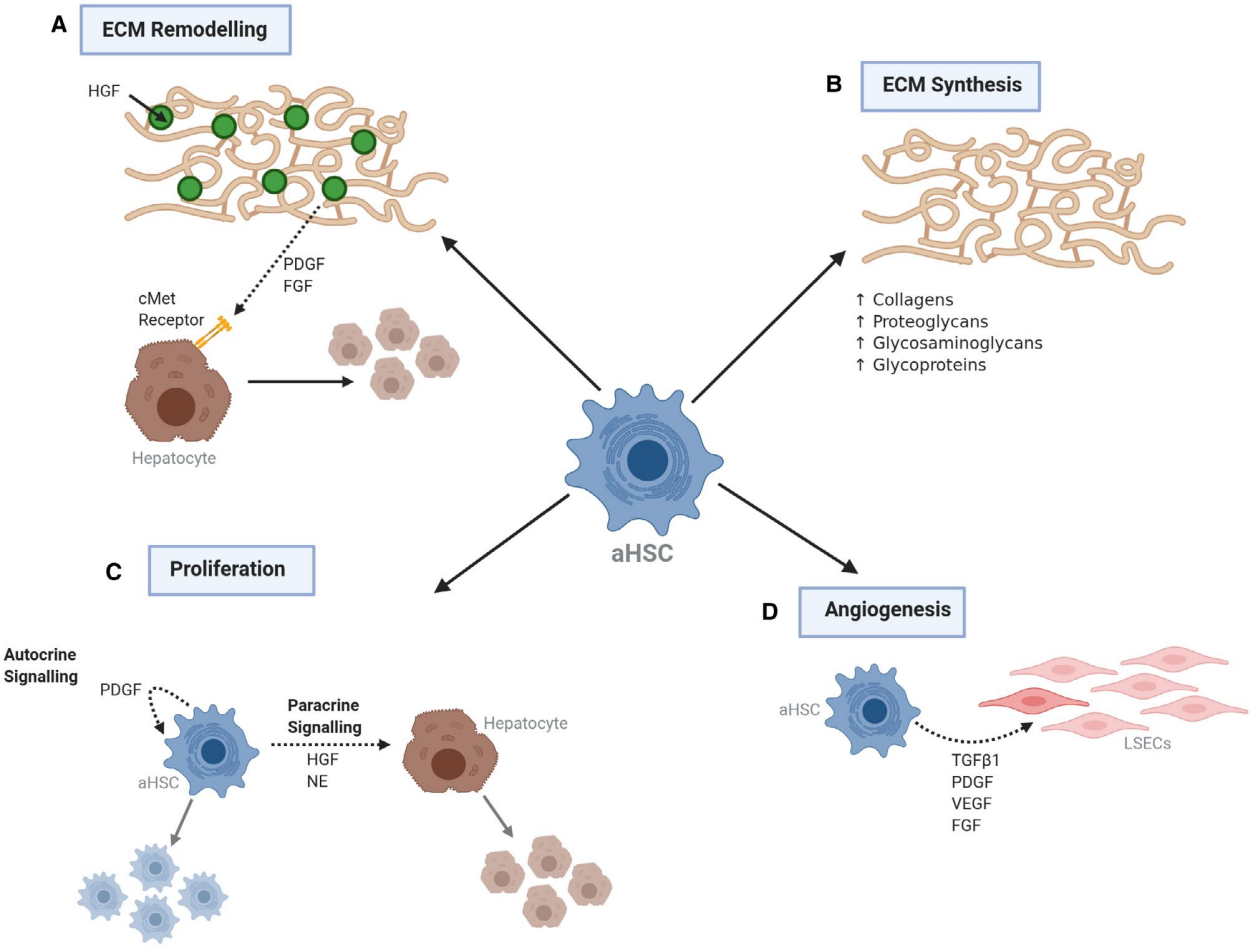
Functional role of HSCs during the hepatic regenerative response. Activated HSCs (aHSCs) have a number of key functions during the hepatic regenerative response. A) aHSCs produce platelet derived growth factor (PDGF) and fibroblast growth factor (FGF) to upregulate the plasminogen system, enabling ECM remodelling and the release of pre‐formed hepatocyte growth factor (HGF) – the primary mitogen for hepatocytes. B) Production of extracellular matrix (ECM) protects against ongoing damage and provides a ‘scaffold’ for repair. C) aHSCs drive regeneration through autocrine and paracrine signalling. Norepinephrine (NE) enhances the mitogenic effect of HGF. D) aHSCs regulate vessel stabilisation and sinusoidal remodelling through direct and paracrine interactions with liver sinusoidal endothelial cells (LSECs). Abbreviations: TGF ß1, transforming growth factor beta 1; VEGF, vascular endothelial growth factor.

Deposition of ECM occurs transiently during the regenerative response, with activated HSCs synthesizing ECM components such as collagens, proteoglycans, glycosaminoglycans, and glycoproteins.^(^ ^65^, ^74^ ^)^ As the primary ECM‐producing cells in the liver, aHSCs generate a temporary scar following injury to protect against further damage.^(^ ^11^ ^)^ Provision of an ECM “scaffold” enables 3D liver growth, supporting the parenchyma and maintaining integrity. Furthermore, HSCs are the main source of matrix metalloproteinases and their inhibitors, which participate in ECM remodeling.^(^ ^75^, ^76^ ^)^ ECM remodeling and cytokine production are closely coupled: HSCs produce PDGF and FGF to up‐regulate the plasminogen system, releasing preformed HGF from the matrix and cleaving active HGF from its inactive form. FGF gene expression increases markedly within the first 24 hours following PHx, and its expression is limited only to HSCs.^(^ ^48^ ^)^ Although ECM deposition has a clear role in the regenerative response following acute injury, in the case of chronic, repeated injury, ongoing ECM accumulation leads to fibrosis and distortion of normal liver architecture. The functional roles of HSCs in the regenerative response have been summarized in Figure 3.

## Termination of Liver Regeneration

Most of the literature in the field has focused on the events during the initiation of regeneration and far less on the pathways leading to its termination. It is clear that HSCs exert both positive and negative influences on the regenerating liver: initially stimulating hepatocyte proliferation through the production of a wide range of growth factors and cytokines, and later generating factors to help curb hepatocyte DNA synthesis when liver mass has been restored.^(^ ^48^ ^)^

TGF‐β is a potent inhibitor of cell proliferation, and for this reason it has been postulated to be an important candidate in the termination of the regenerative response. TGF‐β treatment of rat hepatocytes *in vitro* inhibits DNA synthesis in a dose‐dependent manner.^(^ ^77^ ^)^ *In vivo*, stimulation of 5‐HT_2B_ receptors on the surface of HSCs by serotonin activated the expression of TGF‐β1 by HSCs, which suppressed hepatocyte proliferation through signaling by mitogen‐activated protein kinase 1 and the transcription factor JunD.^(^ ^78^ ^)^ Selective antagonism of 5‐HT_2B_ in models of acute and chronic liver injury enhanced hepatocyte proliferation, providing further evidence for the role of HSC‐derived TGF‐β in termination of the regenerative response. A similar phenotype was seen in 5‐HT_2B_ knockout (KO) mice after PHx.

Studies using transgenic mice with hepatocyte‐specific disruption of the TGF‐β‐II receptor have dissected some of the complexities of TGF‐β signaling in the termination phases of regeneration. Although liver regeneration after PHx is faster in TGF‐β‐II receptor KO mice (TβIIr‐KO), by 120 hours after PHx both wild‐type and TβIIr‐KO mice show decreased cell proliferation.^(^ ^79^ ^)^ Inhibition of Activin A (a member of the TGF‐β superfamily) with follistatin prolonged the proliferation phase in TβIIr‐KO mice,^(^ ^79^ ^)^ suggesting that the TGF‐β‐Activin A complex may have an important role in termination of regeneration.

At the start of the regenerative process, enzymatic degradation of the ECM leads to the release of matrix‐bound growth factors, which drive proliferation. In the termination phases of the regenerative response, reconstitution of the ECM by HSCs allows sequestering of excess growth factors (i.e., HGF and FGF), prompting hepatocytes to exit the cell cycle and return to quiescence.^(^ ^64^ ^)^ This is demonstrated *in vitro*, where the addition of ECM preparations (collagen gels, Matrigel) to hepatocytes in culture inhibits proliferation in response to HGF and EGF and encourages maintenance of a differentiated phenotype.^(^ ^80^ ^)^ Signaling between the ECM and hepatocytes is also important and is mediated by integrins and their associated integrin‐proximal adhesion molecules.^(^ ^81^ ^)^ The integrin‐linked kinase (ILK) signaling complex is activated by interaction with integrins present in the ECM^(^ ^82^ ^)^ and transmits hepatocyte growth suppressor and differentiation enhancement signals.^(^ ^83^ ^)^ Hepatocyte‐specific ILK‐KO mice regenerate their livers significantly faster following PHx, but failure of the termination phase of the regenerative response results in hepatomegaly (liver size 158% of original) 14 days after PHx.^(^ ^83^ ^)^

### HSC FATE DURING TERMINATION OF LIVER REGENERATION

Removal and deactivation of aHSCs are important regulatory mechanisms in the re‐establishment of homeostasis and normal liver architecture. The fate of the mesenchymal cell population following cessation of injury has been studied primarily in the context of fibrosis resolution. It was originally thought that, following resolution of injury, aHSCs/MFBs became senescent or underwent apoptosis.^(^ ^84^ ^)^ In support of this theory, pharmacological induction of apoptosis of aHSCs/MFBs has been shown to accelerate fibrosis resolution.^(^ ^85^ ^)^ However, more recently, studies in mice have shown that aHSCs can also revert to an inactive state, distinct from quiescent HSCs in the uninjured liver and more responsive to subsequent injurious stimuli.^(^ ^84^ ^)^ Inactive HSCs are characterized by down‐regulation of fibrogenic gene expression (Col1a1 [collagen type I alpha 1 chain], α‐SMA, and TIMP1 [tissue inhibitor of metalloproteinase 1]) without up‐regulation of other quiescence‐related genes (Adfp [antibody to adipophilin], Adipor1 [adiponectin receptor 1], and Gfap [glial fibrillary acidic protein])^(^ ^84^ ^)^ (Fig. 2). Troeger et al.^(^ ^86^ ^)^ demonstrated that almost the entire HSC population was activated following injury, with deactivation of a proportion during fibrosis resolution. Using single‐cell polymerase chain reaction, they identified a gradual reduction of HSC activation markers (Col1a1 and TIMP1) in virtually all HSCs over the recovery period. This was confirmed using genetic cell fate tracking in mTom‐mGFP Vim‐CreER mice, in which, despite normalization of fibrosis parameters, mGFP (vimentin) expression persisted in HSCs and colocalized with desmin—suggesting that 40% of aHSCs/MFBs had deactivated.

It is unclear as to why some HSCs persist in a senescent or inactive state, while some undergo apoptosis,^(^ ^84^ ^)^ although studies have suggested that the up‐regulation of pro‐survival signals, such as induction of heat shock proteins,^(^ ^87^ ^)^ may play a key role. In support of this theory, genetic ablation of Hspa1a/b (two members of the Hsp70 family of heat‐shock proteins) increases the susceptibility of aHSCs to gliotoxin^(^ ^85^ ^)^ and tumor necrosis factor‐α^(^ ^88^ ^)^‐induced apoptosis in culture.

## Challenges and Future Directions

The liver is unique in its regenerative capacity, and is the only solid organ that harnesses regenerative mechanisms to maintain a stable organ‐to–body weight ratio, enabling a return to homeostasis following injury. Although other solid organs adjust to tissue loss, they do not return to a normal organ‐to–body weight ratio.^(^ ^89^ ^)^ Although there has been remarkable progress in deepening our understanding of the pathways regulating liver regeneration, the role of the hepatic mesenchyme in this process remains relatively unknown. A comparison of single‐cell‐level mesenchymal data from regenerating livers versus other solid organs (e.g., lung, kidney) following injury could allow an investigation of whether specific subcompartments of the hepatic mesenchyme are intrinsically more pro‐regenerative than the mesenchymal subpopulations identified in other organs, perhaps contributing to the unique regenerative capacity of the liver.

To fully characterize the functional role of HSCs during the initiation, maintenance, and termination of liver regeneration, systems that allow efficient ablation of different HSC subpopulations at different time points in the regenerative process are required.^(^ ^11^ ^)^ As our knowledge of the spatial and functional heterogeneity of HSC during injury and regeneration increases,^(^ ^5^ ^)^ future HSC ablation systems may allow specific targeting of HSC subpopulations during the regenerative process, further deepening our understanding of how the various HSC subclasses regulate liver regeneration. Although it is clear that there is functional heterogeneity in the hepatic mesenchymal response to fibrosis, scRNA‐seq studies using regenerative models, such as PHx, are required to interrogate whether a similar degree of mesenchymal functional heterogeneity exists during liver regeneration.

It has now become clear that hepatic mesenchymal cells play key roles in both the initiation and termination of liver regeneration. Although previous studies have already illuminated some of the mechanisms that HSCs use to regulate the hepatic regenerative response, many unanswered questions remain. In particular, new technologies such as single‐cell genomics will allow further dissection of the various mesenchymal subpopulations regulating liver regeneration, enabling both the precise identification of key, pro‐regenerative mesenchymal subpopulations and the development of highly targeted therapies for patients with liver disease.
